# Supplementary material for: Information and fitness in two-state systems: self-replicating individuals in a fluctuating environment
Source: ArXiv. 2025 Jul 31:arXiv:2508.00150v1. Preprint. [Version 1] (PMC12324550)
Supplement: Supplement 1 [file NIHPP2508.00150v1-supplement-1.pdf]

# Supplementary Information

## S1. DERIVATION OF FRACTION RATE EQUATION

Let $N_{y_j}$ be the number of individuals of phenotype $y_j$ in environment $x_i$, $\in \mathbb{R}^+$; $i, j \in \{1, 2\}$. $N_{y_j}$ is growing exponentially at rate $g_{y_1}^{x_i}$. Assuming that $N_{y_j}$ is high enough, its dynamics can be described by continuous differential equations in a given environmental state $x_i$ as follows:

$$\frac{dN_{y_1}}{dt} = g_{y_1}^{x_i} N_{y_1} - k_{y_1 y_2}^{x_i} N_{y_1} + k_{y_2 y_1}^{x_i} N_{y_2}, \tag{S1a}$$

$$\frac{dN_{y_2}}{dt} = g_{y_2}^{x_i} N_{y_2} + k_{y_1 y_2}^{x_i} N_{y_1} - k_{y_2 y_1}^{x_i} N_{y_2}, \tag{S1b}$$

$$\frac{d(N_{y_1} + N_{y_2})}{dt} = g_{y_1}^{x_i} N_{y_1} + g_{y_2}^{x_i} N_{y_2}. \tag{S1c}$$

In a fixed environment $x_i$, we define $f_{y_j}$ as the fraction of individuals exhibiting phenotype $y_j$ relative to the total population size. Specifically, this implies $f_{y_1} = \frac{N_{y_1}}{N_{y_1}+N_{y_2}}$. We proceed to derive an explicit expression for the time derivative $\frac{d}{dt}\left(\frac{N_{y_1}}{N_{y_1}+N_{y_2}}\right)$.

$$\frac{df_{y_j}}{dt} = \frac{d}{dt}\left(\frac{N_{y_1}}{N_{y_1} + N_{y_2}}\right) = \frac{1}{N_{y_1} + N_{y_2}} \frac{dN_{y_1}}{dt} - \frac{N_{y_1}}{(N_{y_1} + N_{y_2})^2} \frac{d(N_{y_1} + N_{y_2})}{dt} \tag{S2}$$

Using the system of equations (S1a) for the first term and (S1c) for the second term, we get:

$$\frac{d}{dt}\left(\frac{N_{y_1}}{N_{y_1} + N_{y_2}}\right) = \frac{(g_{y_1}^{x_i} N_{y_1} - k_{y_1 y_2}^{x_i} N_{y_1} + k_{y_2 y_1}^{x_i} N_{y_2})}{N_{y_1} + N_{y_2}} - \frac{N_{y_1}(g_{y_1}^{x_i} N_{y_1} + g_{y_2}^{x_i} N_{y_2})}{(N_{y_1} + N_{y_2})^2} \tag{S3}$$

Using the fraction expressions of $f_{y_2} = 1 - f_{y_1} = \frac{N_{y_2}}{N_{y_1}+N_{y_2}}$ and $f_{y_1} = \frac{N_{y_1}}{N_{y_1}+N_{y_2}}$, and simplifying, we obtain,

$$\frac{df_{y_1}}{dt} = k_{y_2 y_1}^{x_i}(1 - f_{y_1}) - k_{y_1 y_2}^{x_i} f_{y_1} + (g_{y_1}^{x_i} - g_{y_2}^{x_i}) f_{y_1}(1 - f_{y_1}) \tag{S4}$$

Similarly, calculating for the phenotype $y_2$, we will get the generalized equation in (1).

## S2. DERIVATION OF NUMERATOR OF Γ

To find the numerator of $\Gamma$ which is $\gamma - \langle\gamma\rangle_{ind}$, we used the equations (8) and (10), and obtain the following,

$$\langle\gamma\rangle - \langle\gamma\rangle_{ind} = g_{y_1}^{x_1} P_{x_1 y_1} + g_{y_2}^{x_1} P_{x_1 y_2} + g_{y_1}^{x_2} P_{x_2 y_1} + g_{y_2}^{x_2} P_{x_2 y_2} - g_{y_1}^{x_1} P_{x_1} P_{y_1} - g_{y_2}^{x_1} P_{x_1} P_{y_2} - g_{y_1}^{x_2} P_{x_2} P_{y_1} - g_{y_2}^{x_2} P_{x_2} P_{y_2} \tag{S5}$$

After using the relation $P_{x_i} = \sum_{j=1}^{2} P_{x_i y_j}$, the expression becomes,

$$\langle\gamma\rangle - \langle\gamma\rangle_{ind} = g_{y_1}^{x_1}(P_{x_1 y_1} - P_{x_1} P_{y_1}) + g_{y_2}^{x_1}((P_{x_1} - P_{x_1 y_1}) - P_{x_1} P_{y_2}) + g_{y_1}^{x_2}((P_{x_2} - P_{x_2 y_2}) - P_{x_2} P_{y_1}) + g_{y_2}^{x_2}(P_{x_2 y_2} - P_{x_2} P_{y_2}) \tag{S6}$$

By rearranging in terms of probability, we can effectively express the differences in growth rates,

$$\langle\gamma\rangle - \langle\gamma\rangle_{ind} = (g_{y_1}^{x_1} - g_{y_2}^{x_1}) P_{x_1 y_1} + (g_{y_2}^{x_2} - g_{y_1}^{x_2}) P_{x_2 y_2} - g_{y_1}^{x_1} P_{x_1} P_{y_1} + g_{y_2}^{x_1}(P_{x_1} - P_{x_1} P_{y_2}) - g_{y_2}^{x_2} P_{x_2} P_{y_2} + g_{y_1}^{x_2}(P_{x_2} - P_{x_2} P_{y_1}) \tag{S7}$$

Using $g_1 = g_{y_1}^{x_1} - g_{y_2}^{x_1}$ and $g_2 = g_{y_2}^{x_2} - g_{y_1}^{x_2}$ the expression of relative growth rates and using the relation $P_{y_j} = \sum_{i=1}^{2} P_{x_i y_j}$ we get,

$$\langle\gamma\rangle - \langle\gamma\rangle_{ind} = (g_{y_1}^{x_1} - g_{y_2}^{x_1}) P_{x_1 y_1} + (g_{y_2}^{x_2} - g_{y_1}^{x_2}) P_{x_2 y_2} - g_{y_1}^{x_1} P_{x_1} P_{y_1} + g_{y_2}^{x_1} P_{x_1} P_{y_1} - g_{y_2}^{x_2} P_{x_2} P_{y_2} + g_{y_1}^{x_2} P_{x_2} P_{y_2} \tag{S8}$$

Simplifying and using the relative growth equation, we get

$$\langle\gamma\rangle - \langle\gamma\rangle_{ind} = (g_{y_1}^{x_1} - g_{y_2}^{x_1}) P_{x_1 y_1} + (g_{y_2}^{x_2} - g_{y_1}^{x_2}) P_{x_2 y_2} - (g_{y_1}^{x_1} - g_{y_2}^{x_1}) P_{x_1} P_{y_1} - (g_{y_2}^{x_2} - g_{y_1}^{x_2}) P_{x_2} P_{y_2} \tag{S9}$$

Further simplification gives us,

$$\langle\gamma\rangle - \langle\gamma\rangle_{ind} = g_{x_1}(P_{x_1 y_1} - P_{x_1} P_{y_1}) + g_{x_2}(P_{x_2 y_2} - P_{x_2} P_{y_2}) \tag{S10}$$

## S3. ANALYTICAL DERIVATION OF THE EXPRESSION FOR FRACTION AND INTEGRATION OF THE PHENOTYPE FRACTION

For the system mentioned in subsection II D, using the growth rates and switching rates, the system equation becomes

$$\frac{df_y}{dt} = k(1-f_y) - kf_y + gf_y(1-f_y) \tag{S11}$$

The fraction $f_y$ denotes the fraction of the fittest phenotype in a given environment. A phenotype is considered "fittest" when it exhibits the highest growth rate among the two phenotypes, $y_1$ and $y_2$, in their corresponding environments, $x_1$ or $x_2$. So $g$ is positive when the phenotype aligns with their optimal environment and negative when it does not. To proceed with solving the equation, we observe that the right-hand side of the equation is a quadratic expression. This allows us to rewrite it as the product of two factors based on its roots. Taking the root as $f_1$ and $f_2$, we can express the equation in terms of these roots:

$$f_1 = \frac{g - 2k - \sqrt{(g^2 + 4k^2)}}{2g}, \tag{S12}$$

$$f_2 = \frac{g - 2k + \sqrt{(g^2 + 4k^2)}}{2g} \tag{S13}$$

Using this and rearranging (S11), we obtain:

$$\frac{df_y}{dt} = g(f_y - f_1)(f_y - f_2) \tag{S14}$$

We analytically derive the expression for the fraction of the population considering a time interval of duration $\Delta t_i$. Here, time $t = 0$ denotes the moment the system enters a specific environment, and time $t = \Delta t_i$ corresponds to the instant just before the system leaves that environment. The resulting expression characterizes the fraction of the population after evolving in a particular environment for a period of time $\Delta t_i$.

$$\begin{aligned} \int_0^{\Delta t_i} \frac{1}{(f_y - f_1)(f_y - f_2)}\, df_{y_j} &= \int_0^{\Delta t_i} (g)\, dt \\ \Rightarrow \int_0^{\Delta t_i} \frac{1}{f_1 - f_2}\left(\frac{1}{f_y - f_1} - \frac{1}{f_y - f_2}\right) df_y &= \int_0^{\Delta t_i} (g)\, dt \\ \Rightarrow \frac{1}{f_1 - f_2} \ln\left(\frac{(f_y(\Delta t_i) - f_1)(f_y(0) - f_2)}{(f_y(\Delta t_i) - f_2)(f_y(0) - f_1)}\right) &= g\Delta t_i \end{aligned} \tag{S15}$$

Solving for $f_y$, the expression for the fraction can be derived in the following form:

$$f_y(\Delta t_i) = \frac{a + ce^{-b\Delta t_i}}{d + he^{-b\Delta t_i}} \tag{S16}$$

where

$$a = 2k - f_y(0)(g + 2k) + f_y(0)\sqrt{g^2 + 4k^2} \tag{S17a}$$

$$b = \sqrt{g^2 + 4k^2} \tag{S17b}$$

$$c = f_y(0)(g + 2k) - 2k + \sqrt{g^2 + 4k^2} \tag{S17c}$$

$$d = (g + 2k) - 2f_y(0)g + \sqrt{g^2 + 4k^2} \tag{S17d}$$

$$h = 2f_y(0)g - (g + 2k) + \sqrt{g^2 + 4k^2} \tag{S17e}$$

The initial condition for $f_y(t)$ is denoted by $f_y(0)$. To compute the integral of $f_y(\Delta t_i)$, we calculate the area under the curve $f_y(\cdot)$ over the time interval $\Delta t_i$,

$$\int_0^{\Delta t_i} f_y(t)\, dt = \frac{abh\Delta t_i}{bdh} - \frac{(cd - ah)}{bdh}\Big(\ln(d + h) - \ln(d + he^{-b\Delta t_i})\Big) \tag{S18}$$

For simplicity, we denote this integral as $\mathcal{I}_y(\Delta t_i)$. Substituting the expressions in eq.(S17) for the parameters, we obtain the following,

$$\mathcal{I}_y(\Delta t_i) = \int_0^{\Delta t_i} f_y(t)\,dt = \frac{(g - 2k + \sqrt{g^2 + 4k^2})\Delta t_i}{2g} + \frac{1}{g}\ln\left(\frac{(2f_y(0) + 2k - g)(1 - e^{-\sqrt{g^2+4k^2}\Delta t_i})}{2\sqrt{g^2 + 4k^2}} + \frac{1 + e^{-\sqrt{g^2+4k^2}\Delta t_i}}{2}\right) \tag{S19}$$

This integral is used to calculate the time-averaged statistics of the joint distributions between phenotypes and environments. The total integration for the fittest phenotype $y$ is given by

$$\mathcal{I}_y(t) = \sum_{i=1}^{n} \mathcal{I}_y(\Delta t_i) \tag{S20}$$

where each $\Delta t_i$ represents a time interval. This cumulative measure can then be used to derive the corresponding probability distribution.

## S4. SIMULATION FOR FIG.6

This algorithm calculates the evolution of the population fraction and therefore the information and fitness of a population of individuals expressing either two phenotypic states, subjected to an environment that fluctuates randomly between a stress condition and a normal growth condition. Specifically, our system considers and environment environmental switching rates $\lambda_{12}$ and $\lambda_{21}$ and therefore the environmental distribution $P_{x_1}$ and $(P_{x_2} = 1 - P_{x_1})$ and phenotypes with distribution $P_{y_1}$. Given the relative growth rates $\mu_1$ and $\mu_2$, we aim to calculate the average population fitness $\langle\gamma\rangle$, the independent population fitness $\langle\gamma\rangle_{\text{ind}}$, the normalized population fitness $\Gamma$, and the mutual information $I$.

The simulation is performed for given values of $P_{x_1}$, $\lambda_{21}$ and $\mu_1$. For each pair $P_{x_1}$, $\lambda_{21}$, the corresponding environmental switching rate from stress ($x_1$) to normal ($x_2$), denoted $\lambda_{12}$, is calculated. The simulation begins by initializing the population's phenotype as the sensitive type $y_1$, with its fraction $f_s$ drawn from an uniform distribution between 0 and 1. The environment is then randomly set to either the proliferating or stress state.

The simulation then proceeds for $N$ iterations or environmental transitions. At each iteration, the time until the environment switches again is sampled from an exponential distribution parameterized by the current environment's switching rate (e.g. $\lambda_{12}$). If the current environment is stressful, the algorithm evaluates how much time within this interval the population spends in each phenotype state by applying an indicator function and updates the counters accordingly.

When the system is in a stress environment, the algorithm updates the phenotypic distribution using a phenotype evolution function, denoted by $f_{y_1}(\Delta t; \mu_1)$, which follows the solution (S16). In this context, the dormant phenotype, $y_1$ is the fittest for the environment $x_1$, and therefore $g$ is assigned a positive value. This function describes how the fraction of individuals in the phenotype $y_1$ evolves over a time interval $\tau$, given the initial condition $f_s$, the death rate $\mu_1$ in the stress environment, and the switching rate, $k$ between phenotypes. In addition to this point-wise update, the algorithm calculates the integral $\mathcal{I}_{y_1}(\Delta t; \mu_1)$, which represents the integration of the fraction of the phenotype $y_1$ during the time spent in the stress environment (S19). After these updates are performed, the environment switches from $x_1$ to $x_2$, and a similar set of operations is carried out using the corresponding function and integral for the normal environment.

A similar series of procedures is followed in the $x_1$ environment: the time spent in that environment is sampled, and the fractions and integrals are updated. The environment then switches back to the stress state. At each iteration, the total simulation time is incremented by the duration $\Delta t$ of the current environmental state.

After completing all iterations, the algorithm normalizes the time duration of each environmental phenotype by the total simulation time to obtain joint probability distributions of phenotype-environment pairs $P_{x_i y_j}$. Marginal probabilities for each phenotype $P_{y_j}$ and each environment state probabilities $P_{x_i}$ are then calculated by summing over appropriate joint probabilities, $\hat{\mathcal{I}}^t_{x_1,y_1}$. These probabilities form the basis for computing key quantities: the average population fitness $\langle\gamma\rangle$, the independent population fitness $\langle\gamma\rangle_{ind}$, the normalized population fitness $\Gamma$, and environmental states; and the mutual information $I$ as explained in the main text.

Finally, the collected data enable a comprehensive analysis of how dormant phenotype switching, environmental stochasticity, and stress-induced death rates interact to shape various population fitness measures and mutual information, as illustrated in the accompanying plots Fig. 6.

**Input:**
$\lambda_{21}$: rate of switching from $x_1$ and $x_2$ (normal to antibiotic environment)
$\mu_2$: growth rate in non-stressful environment
$k$: switching rate between phenotypes
$P_{x_1}$: Stressful environment probability
$\mu_1$: Death rate in stress environment
$N$: number of iterations per simulation
**Output:**
Simulation statistics including:
$P_{x_1}, P_{x_2}, P_{y_1}, P_{y_2}, P_{x_1y_1}, P_{x_1y_2}, P_{x_2y_1}, P_{x_2y_2}, P, \langle\gamma\rangle_{ind}, \langle\gamma\rangle, \Gamma, I$
Compute $\lambda_{12} = \lambda_{21} \cdot \frac{1-P_{x_1}}{P_{x_1}}$
Compute $P_2 = \lambda_{12}/(\lambda_{12} + \lambda_{21})$
Initialize phenotype fraction $f_s \sim \mathcal{U}(0,1)$
Initialize `env` $\sim$ Uniform$\{x_1, x_2\}$
Set total time $t = 0$
Initialize counters: $\hat{P}_{11}^t, \hat{P}_{12}^t, \hat{P}_{21}^t, \hat{P}_{22}^t = 0$
**for** $i = 1$ **to** $N$ **do**
  **if** $env = 1$ **then**
    Sample $\Delta t_i \sim$ Exponential$(1/\lambda_{12})$
    $\hat{Z}_{11} += \mathcal{I}_{y_2}(\Delta t_i; \mu_1)$
    $\hat{Z}_{12} += \Delta t_i - \mathcal{I}_{y_2}(\Delta t; \mu_1)$
    $f_s = f_{y_2}(\Delta t_i; \mu_1)$
    Switch to $env = 2$
  **else**
    Sample $\Delta t_i \sim$ Exponential$(1/\lambda_{21})$
    $\hat{Z}_{21} += \mathcal{I}_{y_2}(\Delta t_i; -\mu_2)$
    $\hat{Z}_{22} += \Delta t_i - \mathcal{I}_{y_2}(\Delta t_i; -\mu_2)$
    $f_s = f_{y_2}(\Delta t_i; -\mu_2)$
    Switch to $env = 1$
  **end**
  $t += \Delta t$
**end**
Distributions:

$$P_{x_i y_j} = \frac{\hat{Z}_{ij}^t}{t}, \quad \text{for } i, j \in \{1, 2\}$$

Marginal Distributions:

$$P_{x_1} = P_{x_1y_1} + P_{x_1y_2}, \quad P_{x_2} = P_{x_2y_1} + P_{x_2y_2}$$

$$P_{y_1} = P_{x_1y_1} + P_{x_2y_1}, \quad P_{y_2} = P_{x_1y_2} + P_{x_2y_2}$$

Growth Rates and Information:

$$\gamma = -\mu_1 \cdot P_{x_1y_2} + \mu_2 \cdot P_{x_2y_2}$$

$$\gamma_{ind} = -\mu_1 \cdot P_{x_1} \cdot P_{y_2} + \mu_2 \cdot P_{x_2} \cdot P_{y_2}$$

$$\Gamma = \frac{\mu_1(P_{x_1y_1} - P_{x_1}P_{y_1}) + \mu_2(P_{x_2y_2} - P_{x_2}P_{y_2})}{(\mu_1 + \mu_2)P_{x_1}P_{x_2}}$$

$$I = \sum_{i,j} P_{x_iy_j} \log_2 \left( \frac{P_{x_iy_j}}{P_{x_i} \cdot P_{y_j}} \right)$$

Return all computed quantities in a data structure.

**Algorithm 1:** This pseudo-code designed to generate the diagram in Fig 6 for fixed $P_{x_1}$, $\lambda_{21}$ and $\mu_1$. All formulas and notations are used consistently and follow standard conventions throughout the manuscript.

## S5. SIMULATION FOR FIG.2B

This algorithm, used for plotting Fig. 2B, is used for plotting the trajectories of the mean fraction in the fluctuating environment. The system transitions between two environments: $x_1$ (stress environment) and $x_2$ (growth environment), with switching rates $\lambda_{12}$ and $\lambda_{21}$, respectively. The slow-growing phenotypic fraction $f_s$ (corresponding to $y_1$) evolves over discrete time steps $\Delta t_i$ according to the analytical expression $f_{y_1}(t)$, which depends on the death or growth rate ($\mu_1$ in $x_1$, $-\mu_2$ in $x_2$) and the phenotypic switching rate $k$. At each step, the environment changes with probability proportional to the product of the rate of the environmental switch $\lambda_{ij}$ and the time step. The current time $t$, the fraction of phenotypes $f_s$, and the state of the environment `env` are stored in an array, which is later converted into a DataFrame for plotting. The resulting trajectory illustrates how the phenotypic composition dynamically responds to stochastic environmental changes, as shown in Fig. 2B.

**Input:**
$\lambda_{12}$: switch rate from environment $x_1$ (antibiotic) to $x_2$ (normal)
$\lambda_{21}$: switch rate from environment $x_2$ to $x_1$
$\mu_1, \mu_2$: growth/death rates in environments $x_1$, $x_2$
$k$: phenotype switching rate
$f_s$: initial phenotype fraction
$\Delta t_i$: fixed time step
$N$: total number of iterations
**Output:**
Time series of $f_s(t)$ and environment `env` used for plotting trajectories
Initialize: $f_s \sim \mathcal{U}(0,1)$, $t = 0$, `env` $\sim$ Uniform$\{x_1, x_2\}$
Initialize empty array `dataarr` = [ ]
**for** $i = 1$ **to** $N$ **do**
  **if** *env* $= x_1$ **then**
    Update phenotype: $f_s \leftarrow f_{y_1}(\Delta t_i; \mu_1)$
    With probability $\lambda_{12} \cdot \Delta t_i$, set `env` $\leftarrow x_2$
  **else**
    Update phenotype: $f_s \leftarrow f_{y_1}(\Delta t_i; -\mu_2)$
    With probability $\lambda_{21} \cdot \Delta t_i$, set `env` $\leftarrow x_1$
  **end**
  $t \leftarrow t + \Delta t_i$
  Append $[t, f_s,$ `env`$]$ to `dataarr`
**end**
Create DataFrame $D$ from `dataarr` with columns: `Time`, `Fraction`, `Env`
**return** *Trajectory plot showing* $f_{y_1}(t)$ *dyanamics with environmental switching*

**Algorithm 2:** Simulate time evolution of phenotype fraction and environment to generate trajectory plot of Fig 2B.
